# Reversible Bending Behaviors of Photomechanical Soft Actuators Based on Graphene Nanocomposites

**DOI:** 10.1038/srep27366

**Published:** 2016-06-06

**Authors:** Dong Niu, Weitao Jiang, Hongzhong Liu, Tingting Zhao, Biao Lei, Yonghao Li, Lei Yin, Yongsheng Shi, Bangdao Chen, Bingheng Lu

**Affiliations:** 1 State Key Laboratory for Manufacturing Systems Engineering, Xi’an Jiaotong University, Xi’an 710049, China

## Abstract

Photomechanical nanocomposites embedded with light-absorbing nanoparticles show promising applications in photoresponsive actuations. Near infrared (nIR)-responsive nanocomposites based photomechanical soft actuators can offer lightweight functional and underexploited entry into soft robotics, active optics, drug delivery, *etc*. A novel graphene-based photomechanical soft actuators, constituted by Polydimethylsiloxane (PDMS)/graphene-nanoplatelets (GNPs) layer (PDMS/GNPs) and pristine PDMS layer, have been constructed. Due to the mismatch of coefficient of thermal expansion of two layers induced by dispersion of GNPs, controllable and reversible bendings response to nIR light irradiation are observed. Interestingly, two different bending behaviors are observed when the nIR light comes from different sides, *i.e.*, a gradual single-step photomechanical bending towards PDMS/GNPs layer when irradiation from PDMS side, while a dual-step bending (finally bending to the PDMS/GNPs side but with an strong and fast backlash at the time of light is on/off) when irradiation from PDMS/GNPs side. The two distinctive photomechanical bending behaviors are investigated in terms of heat transfer and thermal expansion, which reveals that the distinctive bending behaviors can be attributed to the differences in temperature gradients along the thickness when irradiation from different sides. In addition, the versatile photomechanical bending properties will provide alternative way for drug-delivery, soft robotics and microswitches, *etc*.

In the past decade, responsive polymers that can offer external stimuli induced controllable actuations are of fundamental interest^1^. They have represented a novel class of lightweight functional materials enabling new applications in soft robotics^2^, soft actuators^3^, drug delivery^4^, tissue engineering^5^, *etc*. Notably, photoresponsive polymers, due to their advantages in non-contact actuation, remote and local control, omitted connecting wires and electrodes, offer a new method for realizing programmable and reversible mechanical functionalities in photomechanical actuators^6^, optical switches^7^, active optics^8^. Among various photoresponsive polymers, ultraviolet (UV) irradiation induced isomerization and reaction enable the actuations attributed to the size change of azobenzene^8^, spiropyran^9^, diarylethene^10^. However, the applications of UV-responsive actuations will be restricted by the actuation in aqueous solutions and damages of biological tissues from UV light. Alternately, photomechanical nanocomposites, in which light-absorbing nanoparticles are embedded, show promising application in photoresponsive actuations^11^. They will be potentially available in biological systems due to the good penetration of the near-infrared (nIR) light into most biomaterials^12^,^13^. In addition, more universal actuations other than solutions or humidity environments can be accomplished with various soft matrices.

As a branch in photomechanical nanocomposites, the nIR-responsive nanocomposites have attracted great attentions. They are commonly constituted with nIR light absorbing nanomaterials, *i.e.* noble metal nanorods/nanoparticles^14^,^15^, carbon nanotubes^16^ and graphene^17^, with various soft matrices^18^. While graphene shows decreasing absorption from visible to nIR, it shows a brilliant photothermal conversion efficiency in the band of nIR^19^,^20^. When dispersed into polymer matrices, it will absorb and convert nIR light into thermal energy raising the temperature of nanocomposites^21^,^22^. Meanwhile, it also can be incorporated in polymer matrices to modify relevant properties of the nanocomposites due to its brilliant properties^23^,^24^. The photothermal effect combined with the properties modification of nanocomposites makes it an ideal candidate for fabrication new style of photomechanical soft actuators. Some photomechanical actuators based on graphene nanocomposites have been fabricated. Smart nIR driven liquid crystalline elastomers and hydrogels nanocomposites dispersed with graphene are actuated with the thermal effect of graphene^25^,^26^. While the disadvantages of UV are avoided, the fabrication process, response time and aqueous solutions working environments are still necessary to be improved. In addition, photomechanical soft actuators fabricated with thermal plastic nanocomposites dispersing with graphene nanoplatelets (GNPs) have been demonstrated^27^,^28^. However, most mechanical response was constrained by the exerted pre-strains when actuations, which limited the various applications of these GNPs-based photomechanical soft actuators. While impressive of these achievements in photomechanical graphene nanocomposites, it is necessary to develop easily fabricated nanocomposites exploiting the photothermal ability of graphene and accomplishing fast response and widely photomechanical actuations.

Inspired by a bilayer phenomenon that two sheet-like components with different mechanical properties coupled together will attain a shape to facilitate an equilibrium between its constituent elements^29^, a soft actuator is constituted by a bilayer structure. It is composed of Polydimethylsiloxane (PDMS)/graphene-nanoplatelets (GNPs) composited layer (PDMS/GNPs) and pristine PDMS layer. The fabrication procedure is facile and scalable, which only involves scraping coating and spin coating processing for bottom PDMS/GNPs nanocomposites thin layer and pristine PDMS thin layer respectively. Given the brilliant photothermal effect of graphene, and the thermal-expansion mismatch between PDMS and PDMS/GNPs thin layer due to the dispersion of GNPs, controllable and reversible bending response to nIR light irradiation was observed. Interestingly, two distinctive bending behaviors, *i.e.*, gradual single-step bending towards PDMS/GNPs layer when irradiation was from PDMS side, and dual-step bending (bending to the PDMS/GNPs side but with a strong and fast backlash at the time of light is on/off) when irradiation was from PDMS/GNPs side, were observed. In order to understanding the mechanism of the two distinctive bending behaviors, a heat transfer model was established. It reveals that the two distinctive bending behaviors can be attributed to the heat transfer process and the transient temperature gradient along the thickness at the time of irradiation on/off when irradiation from different sides.

## Results

### Design and Fabrication of Soft Bilayers

**Figure 1:**
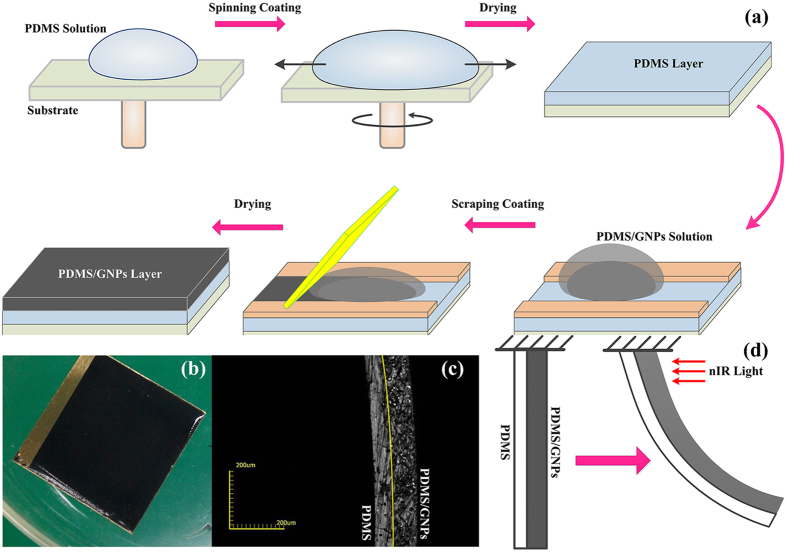
Fabrication and demonstration of the photomechanical soft bilayers. (**a**) Fabrication scheme of soft bilayers. (**b**) Optical image of the soft bilayers. (**c**) Confocal microscopy of sample with 2 wt% GNPs concentration. (**d**) Diagram of bilayer platform actuation. The soft bilayer was vertically anchored on a base and would bend to the PDMS/GNPs side under nIR irradiation.

The fabrication procedure for soft bilayers is facile and scalable, as illustrated in Fig. 1(a), which only involves scraping coating and spinning coating processing for upper PDMS/GNPs nanocomposites layer and bottom pristine PDMS layer respectively. The details of the fabrication process are described in the Methods section. As shown in Fig. 1(b,c), the bilayer structures were demonstrated with the optical image and confocal microscopy respectively. It is observed that the thickness of the bilayer is 130 μm in total, in which the PDMS/GNPs composited layer is 80 μm, and the pristine PDMS is 50 μm. In addition, various GNPs concentrations, which varied from 1 wt% to 5 wt% GNPs concentration, were elected to fabricate soft bilayers. All the samples were cut into strips with the dimension of 10 mm × 1 mm. The soft bilayer was vertically anchored on a base tightly. A nIR light source with the wavelength of 808 nm was chosen to actuate the upper end of the soft bilayers, as shown in Fig. 1(d).

### Photomechanical Bending of Soft Bilayer Actuators

**Figure 2:**
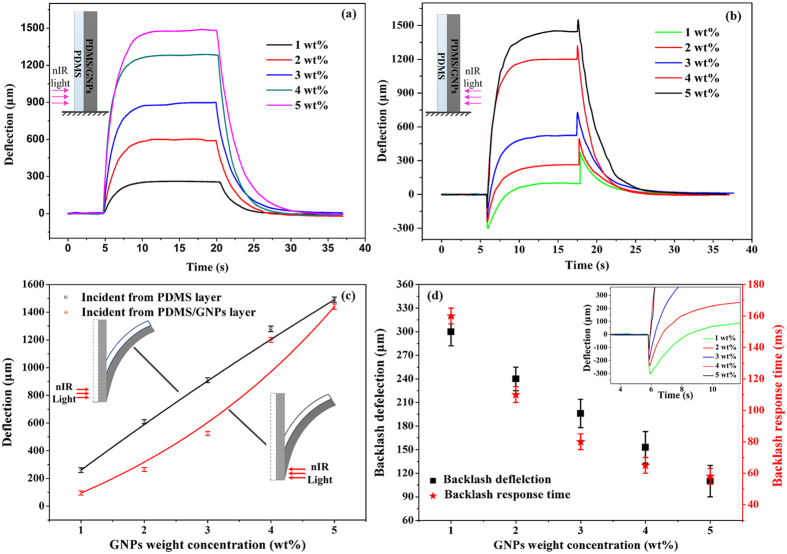
Photomechanical bending behaviors of photomechanical soft bilayer actuators. (**a**) when illuminated from PDMS layer and (**b**) from PDMS/GNPs layer. (**c**) Comparison of photomechanical deflections towards PDMS/GNPs layers, the measurement errors could be obtained with ± 15 μm. (**d**) Backlash deflections and backlash response time when the nIR light is incident from PDMS/GNPs layers, the computed Pearson Correlation coefficient was r = 0.9645, which indicated that the backlash response time displayed highly correlated with backlash deflections.

To observe the photomechanical bending of soft actuators, each layer was illuminated at the end of the soft bilayers by a nIR light source (808 nm) with light intensity with of 2.95 W cm^−2^ respectively. In our previous study, it has revealed that the temperature changes and deflections show an inseparable relationship^30^. It is the temperature change attributed to the photothermal effect of GNPs give rise to the photomechanical bending of the bilayer actuators. In this study, it is further shown that the photomechanical deflection displays positive correlation with the GNPs concentration. Figure 2 shows typical photomechanical bending process when the nIR illumination is incident from each side of the soft bilayer actuators as the light is turned on or off. As illustrated in Fig. 2(a), when the light irradiated from PDMS layer, a reversible single-step bending could be observed. As the light was turned on, all the soft bilayer actuators exhibited continuously photomechanical bending towards the side of PDMS/GNPs nanocomposites. Then it would bend reversely to the initial position as long as the light was turned off, as illustrated in Movie S1, which is in accordance with the common deflection of bilayer actuators. The deflection is enhanced with the augment of GNPs concentration and reached the maximum (about 1500 μm ± 15 μm) at 5 wt% GNPs concentration. In contrast, when the light was incident from the PDMS/GNPs layer, as shown in Fig. 2(b), a dual-step bending was observed. When nIR light was on, the soft bilayer actuators firstly bent fast towards the PDMS side at the time of light on (with a response time in around 100 ms), and then gradually bent towards the PDMS/GNPs side and finally got the steady deflection as the single-step bending described in Fig. 2(a). Upon the removal of the light illumination, a fast bending to PDMS/GNPs layer followed by reversible deflections to the side of PDMS, as illustrated in Movie S2, was also observed. The irregular bending, *i.e.*, the fast bending towards PDMS side at the time of light turned on, and the bending reversely towards PDMS/GNPs side at the time of light turned off, is named by backlash because its bending direction is opposite to that predicted by the bilayer effect. In our experiments, it is observed that the backlash, which occurs at exactly the time of irradiation on or off, shows much faster response (around 100 ms) than the regular bending in Fig. 2(a) (around 3 s). It is also noticed that, the deflections of backlash decrease gradually with the augment of concentration of GNPs, from 350 μm ± 15 μm at 1 wt% GNPs to 110 μm ± 15 μm at 5 wt% GNPs. As shown in Fig. 2(c), it is observed that the photomechanical deflections of these two distinguish bending process are almost the same with 4 wt% and 5 wt% bilayer actuators. However, the final photomechanical deflections towards the side of PDMS/GNPs displays smaller when the nIR light was incident from PDMS/GNPs layer compared to those incident from the PDMS thin layer, which can be attributed to larger backlash deflections at the time of nIR light is on/off. In addition, the backlash deflections and backlash response time also could be extracted from Fig. 2(b). It is noticed that the backlash response time decreases afforded by the addition of GNPs. Correlation coefficient analysis was conducted to determine the strength and direction of the association between backlash deflections and backlash response time. The computed Pearson Correlation coefficient was r = 0.9645, which indicated that the backlash response time displayed highly correlated with backlash deflections, i.e., the backlash response time would increase with the augment of backlash deflections, as the Fig. 2(d) shown.

### Theoretical Models

**Figure 3:**
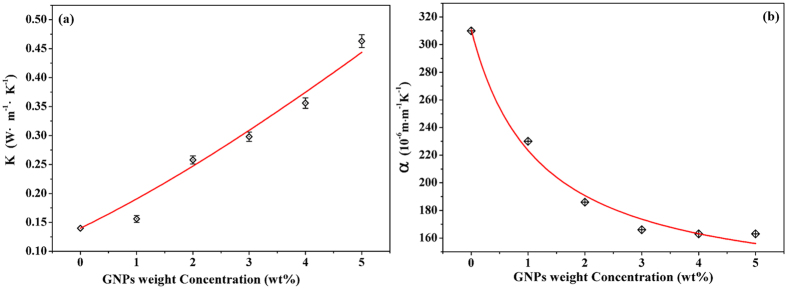
Thermal properties analysis of PDMS/GNPs nanocomposites. (**a**) coefficient of thermal conductivity with various GNPs concentrations at 25 °C. (**b**) coefficient of thermal expansion with various GNPs concentrations at 25 °C.

In our design, the nIR light energy is absorbed by the GNPs existing in the layer of PDMS/GNPs nanocomposites and readily converted to heat, which is then transported to both of the layers. It plays a role in serving as a source of heat in our photoresponsive bilayer actuators. Due to the existence of GNPs, the composited PDMS/GNPs layer takes different thermal properties in contrast to pristine PDMS layer. In order to reveal the heat transfer process, the thermal conductivity (*K*) of the PDMS/GNPs nanocomposites layer was experimentally measured with the hotwire method attributed to the effect it plays a vital role in determining the temperature change of the bilayer actuators. Restricted by the poor thermal conductivity of PDMS (0.15 W∙m^−1^∙K^−1^), the incorporation of high thermally conductive material (*i.e.* GNPs in these bilayer actuators) into PDMS will be suggested to an alternative enhancing the thermal properties^31^. Just as shown in Fig. 3(a), *K* increases with the augment of the GNPs concentration, which can be improved from 0.15 W m^−1^ K^−1^ to 0.45 W m^−1^ K^−1^ at the 5 wt% bilayer actuators. The fitting curve with Geometrical Mean Model^32^ in Note S2 shows a well agreement with the experimental results. Furthermore, the heat transfer process was considered and carried out to obtain the temperature change process in the bilayer actuators. Due to the uniformly and densely distribution of GNPs in PDMS, our bilayers can be considered as a homogeneity materials. The heat transfer equations are considered as shown below:


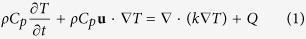



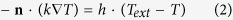


Where *ρ*, *C*_*p*_ and *k* are the density, heat capacity and thermal conductivity, respectively. These equations will be applied into the heat transfer process of each layer. The first term of the right of the Eq. (1) represents the outgoing heat due to the thermal convection with air which is specifically demonstrated in Eq. (2), while the second term denotes the incoming heat due to the photothermal effect of GNPs. The left side of the Eq. (1) is the temperature increase afforded by the heat transfer effect.

Obviously, the coefficient of thermal expansion (*α*) is also the key parameter in our soft bilayer actuators. Earlier studies demonstrated that the incorporation of inorganic fillers with tiny coefficient of thermal expansion (CTE) would have a positive effect on decreasing the CTE of composites^33^. Given the PDMS/GNPs nanocomposites layer in our bilayer actuators, it is reasonable that the CTE of PDMS/GNPs layer will decrease due to the existence of the GNPs, which will cause a deflection towards the layer of PDMS/GNPs just as common phenomenon in bimorph thermal actuators. With the thermal mechanical analysis (TMA) method, the coefficient of thermal expansion (*α*) is experimentally measured. As shown in Fig. 3(b), α of PDMS/GNPs nanocomposites layer will gradually decline to 168 × 10^−6^ m m^−1^ K^−1^ from the 325 × 10^−6^ m m^−1^ K^−1^ of pristine PDMS with the augment of dispersed GNPs weight concentrations. It is obvious that the measured *α* experienced the compliant tendency with the claim in nanocomposites with other inorganic fillers. Considering the Kerner Model^34^, which is proposed to predict the analytical *α* of nanocomposites, a simulated curve was constructed. It is noticed that the Kerner formula in Note S3 can well match the experimental results. In addition, the thermal expansion process has been considered to analyse the bending the bilayer actuators. Due to the poor thermal conductivity of PDMS, the traditionally and commonly utilized equation from S. Timoshenko’s Bimetal Thermostatic Model^35^ based on uniform temperature distribution on both of the two thin layers cannot be applicable in our bilayer actuators. In this paper, a linear elastic thermal expansion equation was adapted to analyse the thermal expansion of each thin layer, as shown below:


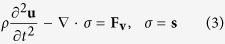



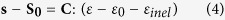



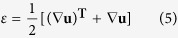



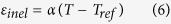


Where *σ* is the Cauchy stress tensor, *ε* is the infinitesimal strain tensor, **u** is the displacement vector, **C** is the fourth-order stiffness tensor, **F**_**v**_ is the body force per unit volume, *T* and *T*_*ref*_ are the temperature obtained in heat transfer process and reference temperature. Equation (3) presents the equation of motion, which is an expression of Newton’s second law. And the Eq. (4) is the Constitutive equations for Hooke’s law. In addition, Strain-displacement equation is depicted in Eq. (5). And the Eq. (6) make a connection betweent heat transfer process and thermal expansion process.

## Discussions

**Figure 4:**
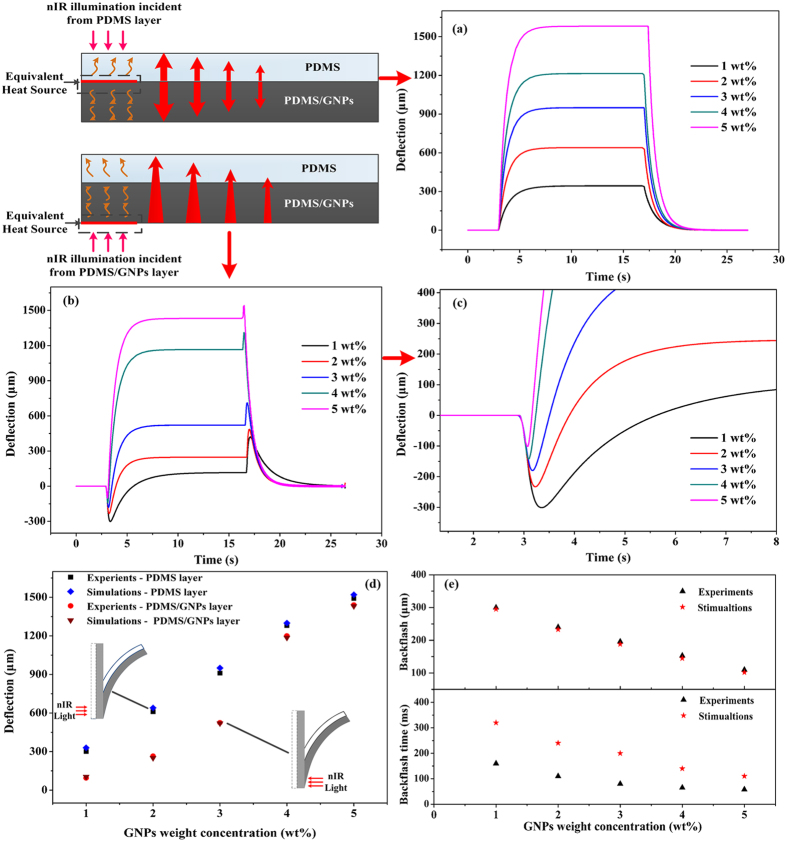
Analytical photomechanical bending process of soft bilayer actuators. (**a**) When illuminated from PDMS layer and (**b**) from PDMS/GNPs layer. (**c**) Analytical backlash of soft bilayers in details. (**d**) Comparisons of photomechanical deflections between experimental measurements and analytical results under two different conditions. (**e**) Comparisons of backlash deflections and backlash response time between experimental measurements and analytical results.

A schematic view of the bilayers is presented in Fig. 4. It consists of PDMS/GNPs thin layer and a pristine PDMS thin layer, which both are anchored and constrained at the left end. These two layers are tightly joined at their interface. A nIR irradiation is considered as a power source and heats over a confined area. The analytical bending of each bilayer actuators with various GNPs weight concentrations will be calculated based on the heat transfer process and thermal expansion process mentioned above. When nIR irradiation was incident from the pristine PDMS layer, nIR light would be absorbed immediately by the GNPs existing at the interface of PDMS and PDMS/GNPs layer, due to the good transparency of pristine PDMS. In this model, an equivalent heat source exerted at the interface was considered. The generated heat will be transferred to both directions from the interface, *i.e.*, PDMS layer and PDMS/GNPs nanocomposites layer. In contrast, an equivalent heat source exerted at the backside of the soft bilayers when the nIR light was incident from PDMS/GNPs nanocomposites layer. It would experience a heat transfer process through the bilayers along the thickness direction from back surface of PDMS/GNPs nanocomposites layer to the upper surface of PDMS layer. Combined the heat transfer process and thermal expansion process, the analytical deflection process was obtained with the *COMSOL Multiphysics*, as shown in Fig. 4(a–c). About the analytical deflections, they experiences almost the same bending process as that in experiments in Fig. 2. The analytical results are then compared to experimental measurements in details. In Fig. 4(d), the photomechanical deflections in these two bending processes are first compared to experimental measurements. There are almost the same deflections towards PDMS/GNPs layer side due to the smaller coefficient of thermal expansion of these layers. In addition, the photomechanical backlash deflection process is also compared with the experiments in terms of backlash deflections and backlash response time. It is noticed that each bilayer actuator will display almost the same backlash deflections between the experimental results and analytical results. It is of significance to analyze the variations between experimental measurements and analytical results in Fig. 4(d,e) with more specific and scientific aspects. First of all, *Two Sample Test for Variance* was conducted to determine whether or not variances of experimental measurements and analytical results are equal. According to the statistics in Table 1 in Note S1, the associated *p-value* in all comparison conditions displayed *p* > *0.05*, which indicated that the variance of experimental measurements and analytical results is not significantly different. Then *Two Sample t Test* was used to test whether or not the means of experimental measurements and analytical results are equal. As shown in Table 2 in Note S1, except for the comparison condition in backlash response time, all the *p-value* displayer *p* > *0.05*, which indicated that the difference of the means of experimental measurements and analytical results is not significantly different. According to the two-step hypothesis easting, it was concluded that analytical results displayed no obvious different with the experimental measurements except for comparison condition in backlash response time. These two facts confirm the effectiveness of our proposed analytical models. However, the simulated backlash response time is higher than the experimental measurements, which could be attributed to that the simulated heat transfer at the beginning of the actuation process cannot precisely demonstrate the practical heat transfer in the soft bilayer actuators.

**Figure 5:**
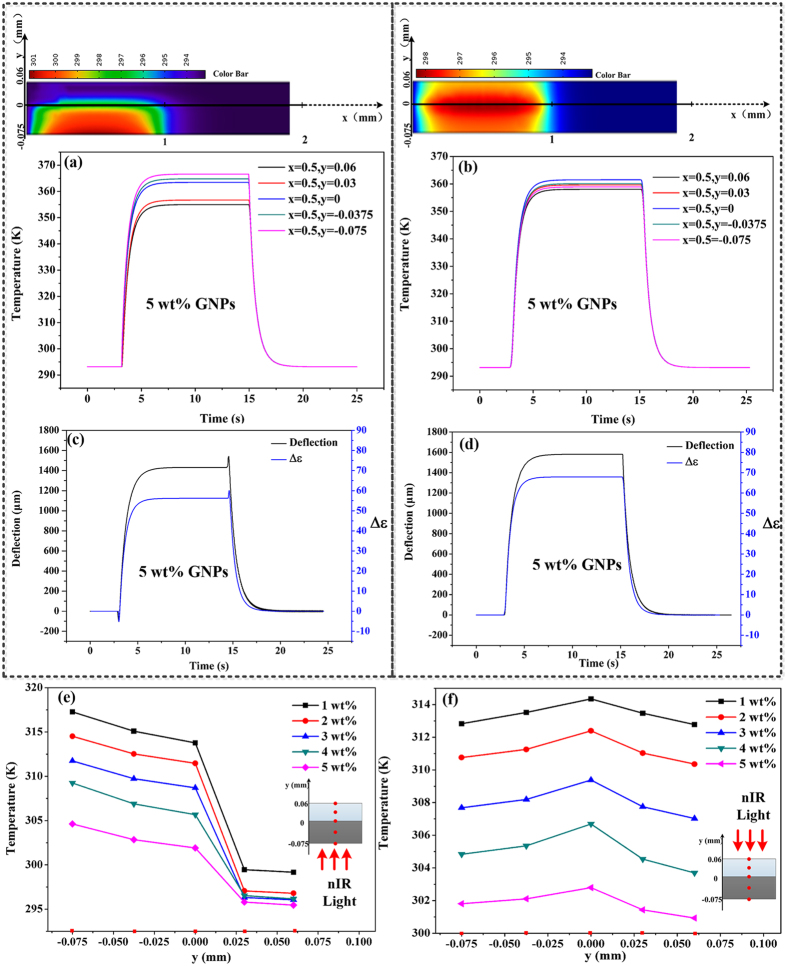
Analytical temperature change along the thickness of the soft bilayers with 5 wt% GNPs concentration. (**a**) When illuminated from PDMS/GNPs layer and (**b**) when illuminated from PDMS layer; Analytical change tendency between Δ*ε* = *εPDMS* − *εPDMS/GNPs* and deflections: (**c**) when illuminated from PDMS/GNPs layer and (**d**) when illuminated from PDMS layer; Analytical temperature gradient along the thickness when the maximum backlash of each soft bilayer was obtained: (**e**) when illuminated from PDMS/GNPs layer and (**f**) when illuminated from PDMS/GNPs layer.

The distinctive bending behaviors, *i.e.* single-step bending process when the light is incident from the PDMS thin layer side, and dual-step bending process when from PDMS/GNPs thin layer side, is further interpreted by the different heat transfer routes and the induced temperature gradients in the two bending processes. The temperature variations along the thickness of soft bilayer actuators during the bending are extracted from the analytical model. For the case that the nIR light is incident from the PDMS/GNPs side, as shown in Fig. 5(a), the heat would transfer from bottom of PDMS/GNPs layer to the PDMS layer. Due to the differences in thermal conductivity of the two layers, a temperature gradient would occur along the thickness of the bilayer actuators when nIR light is on. Because of the difference in thermal expansion for the two layers (*i.e. ε*_*PDMS/GNPs*_ and *ε*_*PDMS*_ for the expansion of PDMS/GNPs and PDMS layer, respectively), Δ*ε* = *ε*_*PDMS*_ − *ε*_*PDMS/GNPs*_ can be obtained, which is responsible for the bending behaviors. As shown in Fig. 5(c) (5 wt% soft bilayers for example), it is obvious that there is a great agreement between the tendency of Δ*ε* and deflection, which indicates the difference in thermal expansion of each layer plays a dominant role in determining the dual-step bending process of the soft bilayers. To further reveal the relationship between deflections and Δ*ε*, a series of representative time point are extracted, as illustrated in Table 1. At exactly the time light is turned on (t_1_), although *α*_*PDMS*_ is larger than *α*_*PDMS/GNPs*_, the thermal expansion of PDMS/GNPs layer (*ε*_*PDMS/GNPs*_) is over the thermal expansion of PDMS (*ε*_*PDMS*_), which is attributed to the higher temperature of PDMS/GNPs layer (because of its larger thermal conductivity than that of PDMS) than that of PDMS layer. In this case, Δ*ε* = *ε*_*PDMS*_ − *ε*_*PDMS/GNPs*_ < 0, and gives rise to a bending towards PDMS layer, (that is the backlash bending behavior), and the backlash deflection gets the maximum at t_2_, where Δ*ε* reaches to the maximum negative value. As the heat transfer continues as light on (t_3_ → t_4_), the *ε*_*PDMS*_ gradually increases and finally gets larger than *ε*_*PDMS/GNPs*_, inducing gradually bending towards the layer of PDMS/GNPs and gets a steady deflection at t_5_. Similarly, at exactly the time the light is turned off, the temperature of PDMS/GNPs layer drops much faster (also because of its much larger thermal conductivity) than that of the PDMS layer, resulting in Δ*ε* enlarged and reaching to the maximum positive value at t_6_, thus the backlash bending towards PDMS/GNPs layer occurs. As the heat convection continues, the heat expansion of each layer will gradually decrease to the initial state (t_7_ → t_8_) induced by the decline of the temperature, thus the bilayer actuator would recover to its initial position at t_9_, where Δ*ε* goes back to 0.

In contrast, when nIR light is incident from PDMS layer, the heat would transfer to both of the two layers from the interface. As shown in Fig. 5(b), there is no obvious temperature gradient occurred along the thickness of the soft bilayer actuator. Obviously, Δ*ε* is monotonic increasing when light is on, and monotonic decreasing when light is off, which is responsible for the single-step bending process, as shown in Fig. 5(d). As shown in Table 2, there exists a continuously Δ*ε* = *ε*_*PDMS*_ − *ε*_*PDMS/GNPs*_ > 0 in the whole period of light on (t_1_ → t_5_) due to the effect of *α*_*PDMS*_ > *α*_*PDMS/GNPs*_, thus the soft bilayer actuator bends towards the PDMS/GNPs layer monotonically. When nIR light is off (t_6_ → t_9_), it would be gradually recovered to its initiate sta

Furthermore, to explore whether this explanation can be applicable for all the soft bilayers with different GNPs concentrations, temperature distributions were extracted at the time of the maximum backlash deflection. Five points were selected at the x = 0.5 mm (the centre of light spot) in the y direction (across the thickness). The soft bilayer actuators with various GNPs concentrations displayed the almost same case in these two different bending processes. As shown in following Fig. 5(e,f), it was clearly noticed that, when the nIR light was incident from PDMS/GNPs layer, the temperature in the layer of PDMS/GNPs (e.g., y = −0.075 mm and y = −0.0375 mm) was higher than that in the layer of PDMS (e.g., y = 0.03 mm and y = 0.06 mm), and the temperature gradually dropped from PDMS/GNPs layer to PDMS layer. The decrease in Δ*ε* would induce a reduction in backlash deflection towards the PDMS/GNPs layer with the augment of GNPs concentrations, which agrees the experimental results. However, when the nIR light was incident from PDMS layer, the temperature along the thickness displayed approximately symmetrical distributions, with a centre of y = 0, which is the interface of the two layers. The temperature at the centre (y = 0) got maximum, and gradually declined towards PDMS/GNPs layer and PDMS layer, respectively. There is no obvious temperature gradient observed in each soft bilayer when the nIR light incidents from PDMS layer, and the larger thermal expansion of PDMS layer (*ε*_*PDMS*_ > *ε*_*PDMS/GNPs*_) is responsible for the single-step bending process.

### Repeatable Photomechanical Bending

**Figure 6:**
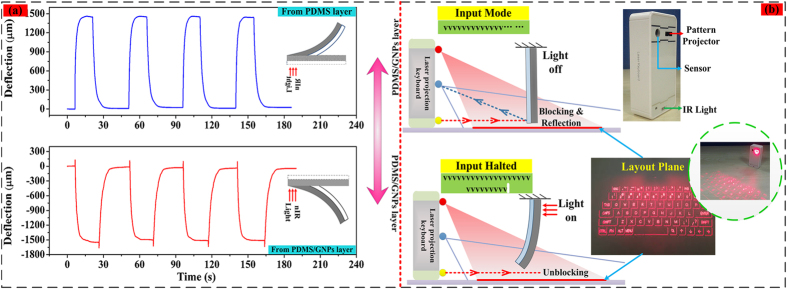
Demonstration of an optical switch. (**a**) The photomechanical deflections under multicycle nIR light when illuminated from different layers and its application in optical switches. (**b**) Diagram of optical switches made from the photomechanical soft actuators with laser projection keyboard as a portable input device.

Bending repeatability, as a key indicator, plays an important role in evaluating the performances of the soft photomechanical actuators. In this section, the photomechanical deflections were measured by multicycle nIR illumination to further demonstrate motion repeatability of our proposed soft bilayers. We took the bilayer sample with 5 wt% GNPs for example, which were illuminated from the PDMS layer and PDMS/GNPS layer, respectively, as Fig. 6(a) indicates. It is shown that our proposed photomechanical actuators both show repeatable photomechanical deflections, which indicates that the soft bilayers are qualified with well repeatability, and promising for soft actuators.

In addition, due to the fast response (in 100 ms level) of the backlash deflection in the dual-step photomechanical bending process, the photomechanical soft actuator supplies an alternative mean for an optical microswitch. In this section, the optical switch is represented as artificial finger to control the input of a laser projection keyboard, as shown in Fig. 6(b). It is known that the laser pattern projector at the top projects visible virtual keyboard onto level surface. Meanwhile, the IR light at the bottom projects an invisible infrared beam above the virtual keyboard. At the time user’s finger makes a keystroke on the virtual keyboard, the invisible infrared beam from IR light will be broken and reflected back to the sensors in the middle of laser projection keyboard. The sensor chip will determine where infrared beam was broken and make the corresponding input. In our experimental demonstration, the optical switch could play a role in altering the working conditions of the laser projection keyboard. At initial state, the invisible infrared beam above the virtual keyboard will be broken and blocked by the soft actuator, and reflected to the sensors of the laser projection keyboard, resulting in a continuous input. As long as the illumination is incident from the PDMS/GNPs layer, the optical switch is turned on due to the backlash towards PDMS layer, therefore, the reflection of the invisible infrared beam above the virtual keyboard is dismissing, resulting in the interruption of input, as demonstrated in details in Movie S3. The response time (from blocking input to non-blocking input) is about 100 ms, greatly improved compared to the most mentioned microswitches with single-step bending process of the photoresponsive soft actuators. Therefore, dual-step bending with a fast backlash of the photomechanical soft bilayer provides an option in developing new remotely controllable and soft microswitches with sub-second response.

## Conclusions

In this article, we present new soft and photomechanical actuators with bilayer structure. Due to the photothermal effect of GNPs and differences in coefficient of thermal expansion of the two thin layers with the addition of GNPs, the soft bilayers can be photomechanical bending under nIR light irradiation. When the soft actuators are illuminated form the side of PDMS thin layer, it would experience a single-step bending towards PDMS/GNPs side. In contrast, a dual-step bending behaviour, *i.e.* fast backlash bending (the response time is in sub-second) at the time of light on/off and gradual bending process at the other illumination time, is observed when the bilayer is irradiated from the PDMS/GNPs side. The two distinctive photomechanical bending behaviors are further interpreted in terms of heat transfer process and thermal expansion process. We found that the temperature gradients induced by different heat transfer along the thickness of the soft bilayers are responsible for the two distinctive bending behaviours. In addition, we further explore the actuation repeatability of the proposed photoresponsive bilayer. We believe that the proposed soft photoresponsive bilayer would find vast applications, such as microcantilevers, microswitches, micro/nanorobotics, drug delivery, active optics, *etc*.

## Methods

### Fabrication of Soft Bilayer Actuators

GNPs were purchased from *JiangNan Graphene Research Institute* and were directly used in their original form. PDMS obtained from *Dow Corning* (*Sylgard 184*) was used as the host matrix. PDMS/GNPs composite was prepared by weighing desired amount of GNPs and adding to the PDMS crosslinker. Then the PDMS base compound was added at a ratio of 10:1 to the PDMS crosslinker and mixed. In addition, the pure PDMS solution was configured by the PDMS base compound and crosslinker at a ratio of 10:1. The fabrication procedure is facile and scalable, which only involves scraping coating and spinning coating processing for upper PDMS/GNPs nanocomposites layer (80 μm in thickness) and bottom pristine PDMS layer (50 μm in thickness) respectively.

### Physical Characterization of the Soft Bilayer Actuator

The GNPs concentrations in this study were from 1 wt% to 5 wt%. All the samples were made into strips with the dimension of 10 mm × 1 mm. A nIR light source with the wavelength of 808 nm was chosen to actuate the soft bilayers. A *KEYENCE* displacement sensor was used to record the tip deflection of soft bilayers when the nIR light is incident from the PDMS and PDMS/GNPs respectively. The measurement of the thermal conductivity was conducted with the thermal analyzer from *TC 3000* series in *XIATECH THW technique*. In addition, with the thermomechanical analysis supported by *NETZSCH TMA 402 F1/F3 Hyperion^®^*, the coefficient of thermal expansion of the soft bilayers was measured.

## Additional Information

**How to cite this article**: Niu, D. *et al.* Reversible Bending Behaviors of Photomechanical Soft Actuators Based on Graphene Nanocomposites. *Sci. Rep.* **6**, 27366; doi: 10.1038/srep27366 (2016).

## Supplementary Material

Supplementary Information

Supplementary Movie S1

Supplementary Movie S2

Supplementary Movie S3

## Figures and Tables

**Table 1:**
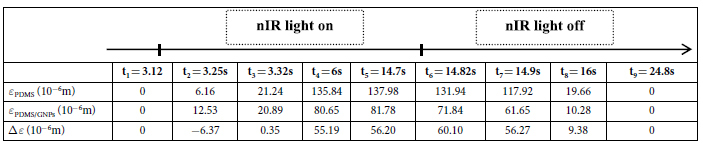
The thermal expansion of PDMS and PDMS/GNPs when the nIR light was illuminated from PDMS/GNPs.

t_2_ and t_6_ are the moment when the maximum backlash deflection is obtained at the time nIR light on and off respectively. t_2_ → t_5_ demonstrates the bending process towards PDMS/GNPs layer. And t_7_ → t_9_ is the bending process recovering to initial state.

**Table 2:**
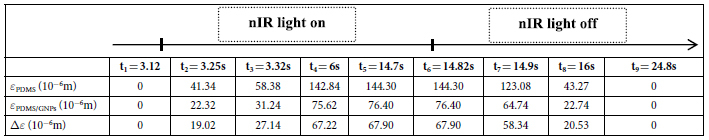
The thermal expansion of PDMS and PDMS/GNPs layer when the nIR light was illuminated from PDMS layer.

t_1_ → t_9_ is the completely bending process from light on to light off.
