# Supplementary material for: Reversible Bending Behaviors of Photomechanical Soft Actuators Based on Graphene Nanocomposites
Source: Sci Rep. 2016 Jun 6;6:27366. doi: 10.1038/srep27366 (PMC4893616; doi:10.1038/srep27366)
Supplement: Supplementary Information [file srep27366-s1.pdf]

Supplementary Information

Reversible Bending Behaviors of Photomechanical Soft Actuators Based on Graphene Nanocomposites

Dong Niu [1], Weitao Jiang [1,*], Hongzhong Liu [1,*], Tingting Zhao [1], Biao Lei [1], Yonghao Li [1], Lei Yin [1], Yongsheng Shi [1], Bangdao Chen [1], Bingheng Lu [1]

[1] State Key Laboratory for Manufacturing Systems Engineering, Xi’an Jiaotong University, Xi’an 710049, China

* wtjiang@mail.xjtu.edu.cn; hzliu@mail.xjtu.edu.cn

Supplementary Notes

Supplementary Notes S1 | Statistics for experimental deflections and simulation results

To verify the feasibility of the simulation model proposed in our article, experimental deflections and simulation results were compared in details, i.e., photomechanical deflections-incident from PDMS layers, photomechanical deflections-incident from PDMS/GNPs layers, backlash deflections and backlash response time. It is of significance to analyze the variations between experimental measurements and simulation results with more specific and scientific aspects. First of all, ***Two Sample Test for Variance*** was conducted to produces an ***F test*** to determine whether the variances of experimental measurements and simulation results are equal. According to the analysis report generated in Table 1, the associated ***p-value (Prob>F)*** in all comparison conditions displayed ***p>0.05***, which indicated that the population variance of experimental measurements and simulation results is not significantly different at the 0.05 level. Then ***Two sample t Test*** was used to test whether the means of experimental measurements and analytical results are equal. With the effect the population variance of experimental measurements and simulation results is not significantly different, it was suitable to choose the ***t-Test statistics*** as ***Equal Variance Assumed*** and obtained the ***t statistic*** and the associated

p-value (Prob>|t|). As shown in Table 2, except for the comparison condition in backlash response time, all the ***p-value (Prob>|t|)*** displayer ***p>0.05***, which indicated that the difference of the population means of experimental measurements and simulation results is not significantly different at the 0.05 level. According to the two-step hypothesis easting, It was concluded that analytical results displayed no obvious different with the experimental measurements except for comparison condition in backlash response time.

Table 1. ***Two sample Test for Variance***

Two Sample Test for Variance	**Deflections-incident from PDMS layers**	**Deflections-incident from PDMS/GNPs layers**	**Backlash deflections**	**Backlash response time**
F Stastic	1.00776	1.0183	0.96956	0.25084
Prob>F	0.9942	0.9864	0.97682	0.20903

*When the $p>0.05$, it is indicated that at the 0.05 level, the two population variance is NOT significantly different; When the $p<0.05$, it is indicated that at 0.05 level, the two population variance is significantly different.

Table 2. Two sample t test

Two Sample t Test	**Deflections-incident from PDMS layers**	**Deflections-incident from PDMS/GNPs layers**	**Backlash deflections**	**Backlash response time**
t Stastic	0.01837	-0.0983	0.15257	-2.58128
Prob>|t|	0.92412	0.9858	0.88251	0.03255

* When the $p>0.05$, it is indicated that at 0.05 level, the difference of the population means is not significantly different with the test difference; When the $p<0.05$, it is indicated that at 0.05 level, the difference of the population means is significantly different with the test difference.

Supplementary Notes S2 | Fitted formula for thermal conductivity

Due to the existence of GNPs, the composited PDMS/GNPs layer takes different thermal properties in contrast to pristine PDMS layer. The incorporation of GNPs into PDMS will be suggested to an alternative enhancing the thermal conductivity. In order to verify the experimental measurements, ***Geometrical Mean Model*** was selected to fit the thermal conductivity (K) with various GNPs concentrations. The formula used for fitting the experimental thermal conductivity (K) of the PDMS/GNPs nanocomposites are as following:

$$\begin{aligned} \varphi &= W \Big/ \left(W + (1 - W) \times \rho_{GNPs} / \rho_{PDMS} \right) \\ K_{PDMS/GNPs} &= K_{GNPs}^{\varphi} \times K_{PDMS}^{(1-\varphi)} \end{aligned} \qquad \textbf{(S1)}$$

Where W, ρ_{GNPs}, ρ_{PDMS}, K_{GNPs} and K_{PDMS} are the GNPs concentration, the density of GNPs and PDMS and coefficient of thermal conductivity of GNPs and PDMS, respectively.

Supplementary Notes S3 | Fitted formula for coefficient of thermal expansion

The incorporation of inorganic fillers with tiny coefficient of thermal expansion (CTE) would have a positive effect on decreasing the CTE of composites. It is reasonable that the coefficient of thermal expansion (α) of PDMS/GNPs nanocomposites will decrease due to the existence of the GNPs. As the experimentally measured, coefficient of thermal expansion (α) of PDMS/GNPs nanocomposites gradually decline with the augment of dispersed GNPs weight concentrations. In order to verify the experimental measurements, ***Kerner Model*** was selected to fit the coefficient of thermal expansion (α) with various GNPs concentrations. The formula used for fitting the experimental coefficient of thermal expansion (α) of the PDMS/GNPs nanocomposites are as following:

$$\begin{aligned} &\varphi = W \Big/ \left(W + \left(1 - W \right) \times \rho_{GNPs} / \rho_{PDMS} \right) \\ &\theta = \left(1/K_{PDMS} - 1/K_{GNPs} \right) \Big/ \left[\varphi / K_{PDMS} - \left(1 - \varphi \right) / K_{GNPs} + 0.75 \times G_{PDMS} \right] \\ &\alpha_{PDMS/GNPs} = \left(1 - \varphi \right) \times \alpha_{PDMS} + \varphi \times \alpha_{GNPs} - \left(\alpha_{PDMS} - \alpha_{GNPs} \right) \times \varphi \times \left(1 - \varphi \right) \times \theta \end{aligned} \qquad \textbf{(S2)}$$

Where W, ρ_{GNPs}, ρ_{PDMS}, K_{GNPs} and K_{PDMS} are the GNPs concentration, the density of GNPs and PDMS and coefficient of thermal conductivity of GNPs and PDMS, respectively.

Supplementary Movies S1-S3

Supplementary Movie S1 | Single-step photomechanical bending process when the soft actuator was illuminated from PDMS layer (with bilayer with 3 wt% GNPs as an example).

Supplementary Movie S2 | Dual-step photomechanical bending process of the photomechanical actuator illuminated from PDMS/GNPs layer (with bilayer with 3 wt% GNPs as an example).

Supplementary Movie S3 | An optical switch with the soft actuator was demonstrated, where continuous input occurred at the nIR light was on, while there was not input as long as the nIR light was turned off.
